# Network Analysis of Plasmidomes: The *Azospirillum brasilense* Sp245 Case

**DOI:** 10.1155/2014/951035

**Published:** 2014-12-29

**Authors:** Valerio Orlandini, Giovanni Emiliani, Marco Fondi, Isabel Maida, Elena Perrin, Renato Fani

**Affiliations:** ^1^Institute of Protein Biochemistry, National Research Council, Via P. Castellino 111, 80134 Naples, Italy; ^2^Laboratory of Microbial and Molecular Evolution, Department of Biology, University of Florence, Via Madonna del Piano 6, 50019 Sesto Fiorentino, Firenze, Italy; ^3^Tree and Timber Institute, National Research Council, Via Madonna del Piano 10, 50019 Fiorentino, Firenze, Italy

## Abstract

*Azospirillum brasilense* is a nitrogen-fixing bacterium living in association with plant roots. The genome of the strain Sp245, isolated in Brazil from wheat roots, consists of one chromosome and six plasmids. In this work, the *A. brasilense* Sp245 plasmids were analyzed in order to shed some light on the evolutionary pathways they followed over time. To this purpose, a similarity network approach was applied in order to identify the evolutionary relationships among all the *A. brasilense* plasmids encoded proteins; in this context a computational pipeline specifically devoted to the analysis and the visualization of the network-like evolutionary relationships among different plasmids molecules was developed. This information was supplemented with a detailed (*in silico*) functional characterization of both the connected (i.e., sharing homology with other sequences in the dataset) and the unconnected (i.e., not sharing homology) components of the network. Furthermore, the most likely source organism for each of the genes encoded by *A. brasilense* plasmids was checked, allowing the identification of possible trends of gene loss/gain in this microorganism. Data obtained provided a detailed description of the evolutionary landscape of the plasmids of *A. brasilense* Sp245, suggesting some of the molecular mechanisms responsible for the present-day structure of these molecules.

## 1. Introduction

*Azospirillum brasilense* is a Gram-negative, aerobic, free-living nitrogen-fixing bacterium found in the soil associated with roots [1, 2]. Several* A. brasilense* strains have been isolated over time. One of the most studied ones, reported as Sp245, was isolated in Brazil in 1986 from surface-sterilized wheat roots where it interacts endosymbiotically with the plant and performs nitrogen fixation [3]. The whole genome sequence of this strain has been recently determined, revealing that it is constituted by seven replicons: one chromosome (accession number NC_016617) and six plasmids (AZOBR1, AZOBR2, AZOBR3, AZOBR4, AZOBR5, and AZOBR6) of different length (accession numbers NC_016594, NC_016618, NC_016595, NC_016596, NC_016619, and NC_016597, resp.), one of them (AZOBR1) being very large, with a size over 1.7 Mb [1, 4]. Interestingly, a comparative genomics analysis revealed that the genus* Azospirillum *might represent an evolutionary bridge between water- and soil-living bacteria [4]. Therefore, the insight into its genes and proteins is important not only to know the role that* Azospirillum* plays in the interaction between plants and bacteria but also to gain a deeper knowledge of the mechanisms that brought bacteria from water to soil environments. Plasmids are particularly intriguing from this viewpoint because of their high variability and the ability to easily exchange genetic material among them and/or with the genome either of the same or of other strains. For these reasons they represent excellent candidates for a research that aims at improving the current knowledge in the fields of* Azospirillum* genomics and phylogenetics. Available studies showed that plasmids evolve primarily through the horizontal gene transfer (HGT) occurring between different strains belonging to the same or to different species/genera, and it is possible to map such exchanges by constructing networks linking the genes of the studied organism with the most similar ones found in the DNA molecules harbored by other strains [5–8]. Therefore, in this context it could be also possible to study the “Preferential Organismal Sharing” (POS), which has been defined as the strongest evolutionary relatedness (based on sequence similarity) of each gene in the replicons with taxonomically and/or ecologically related organisms [9].

The availability of massive database of plasmid sequences, together with the development of bioinformatics tools enabling the visualization of sequence homology relationships through similarity networks [10], can be very useful in the study of the molecular evolution events (e.g., gene duplication and/or gene mobility) either within the same replicon or between replicons of different strains/species or within the same cytoplasm. In particular, the last issue has received much less attention and, to the best of our knowledge, just two examples of deep analysis of plasmids inhabiting the same cytoplasm have been reported and concerned with the* Synechococcus* sp. PCC 7002 [11] and* Burkholderia vietnamiensis* G4 [9]. With the development of network-oriented representation of sequences similarity relationships [6, 10, 12, 13] graph theory measures have been applied to better describe the gene(s) flow across the diverse microbial communities, paving the way to large scale comparative analyses adopting bioinformatics strategies.

In this work a computational network-oriented visualisation and analysis pipeline was developed and used to analyse the evolutionary relationships existing within and among the replicons of* A. brasilense* Sp245 in order to clarify (i) which and how many paralogous genes do plasmids feature, (ii) whether it might be possible to identify any correlation between the number of paralogs and the plasmid size, (iii) how paralogous genes are arranged (tandem or scattered) and organized (stand-alone, clustered, or operonic) onto their plasmid backbone, (iv) which the functions performed by paralogs are (in other words, is there a bias in duplicated genes functions?), (v) if different plasmids have different or similar evolutionary trajectories both at intrareplicon and interreplicon level, and (vi) the extent of HGT with other bacteria.

## 2. Materials and Methods

### 2.1. Analysis of* A. brasilense* Sp245 Plasmidome

The analyses on the* A. brasilense* Sp245 plasmidome (Table 1) were performed through open source bioinformatics tools. A graphical representation of the entire pipeline is reported in Supplementary File 1 available online at http://dx.doi.org/10.1155/2014/951035. To find out the similarities among proteins, the BLAST+ 2.2.27 program [14, 15] was used. We considered as significant those matches with an identity value of at least 40.0%, an *E*-value not greater than 0.05, and the alignment covering not less than the 70% of the query sequence. A C++ utility was written to automatically parse the BLAST output, together with original FASTA file, and return only the significant matches.

The functional characterization of the proteins and the retrieval of the closest orthologs of each query sequence were performed with BLAST2GO [16].

The graphical representation of the plasmids was obtained with Circos [17], whereas the similarity networks were made with Cytoscape [18] and its plugin NetCirChro (available at http://exon.niaid.nih.gov/netcirchro/).

The pipeline that brings from the FASTA sequences file to the formatted table was developed specifically for this work. The programming languages used were Ruby and C++. In detail, the first script performs a BLAST search of the protein sequences against themselves, in order to find possible connections. From the BLAST result, it removes the self-hits and those that do not meet (simultaneously) all the above-mentioned criteria that are user-defined to achieve either lower or higher stringency in the analyses. Finally, the script creates two files, one with the connected sequences (the sequences scoring significant BLAST hits) and the other one with those without relevant hits. These files can be directly used to feed BLAST2GO. The text results of the BLAST online search performed inside BLAST2GO are passed to the second script, which extracts the first hit not belonging to the same strain of the query organism and builds up the table as a tab-separated text file which can be opened by any spreadsheet application or by a simple text editor. The table summarizes the features of the proteins analyzed: GI, function (Gene Ontology annotation), whether it has orthologs or not, organism with the most similar sequence (if any), and, in case, the identity percent and the *E*-value.

### 2.2. Functional Annotation

Functional annotation of each of sequences in the assembled dataset was obtained according to the best BLAST hit in the COG database, with a threshold *e*-value of 1*e* − 20. This annotation was automatically obtained with a custom made script, that we called BLAST2COG.

### 2.3. Identification of the POS

The Preferential Organismal Sharing (POS) has been recently defined as the strongest evolutionary relatedness (based on sequence identity) of each gene of a given replicon with taxonomically and/or ecologically correlated organisms [9].

To find the POS of a gene, an online BLAST search has been performed, using the translated protein sequence of the gene as query, with the software BLAST2GO (available on https://www.blast2go.com/). A custom made script filters the results of BLAST2GO, finding the first BLAST match belonging to a strain other than the one the analyzed gene belongs to.

The organism identified in this way is that yielding the sequence exhibiting the highest degree of sequence similarity with the query one, and it is assumed to be the organism, which probably has an evolutionary (vertical or horizontal) relatedness to the former. This supposition is often supported by the fact that the organisms found as POS have known relationships with the analyzed strain. This relationship can be taxonomical, ecological, or both, with a prominence of the organisms sharing the same niche over the ones taxonomically near but living in a different environment.

## 3. Results

Replicon networks construction and analysis may allow a multilevel observation and analysis of DNA molecules structure and evolution. Such approach was applied here to study the number, the physical organization, and the function of paralogous genes of* A. brasilense* Sp245 plasmid genes at three different levels: (i) intraplasmid level, that is, the molecular evolution events occurring within the same plasmid; (ii) interplasmids level, that is, the evolutionary relatedness among different plasmids; (iii) interplasmid/chromosome level, that is, the cross talk among plasmids and chromosome.

In this analysis, the existence of a gene coancestry is expressed as a link connecting two or more nodes, in case all the sequence identity minimal parameters (described in Section 2) are met. According to Maida et al. [9], the analysis of these networks might allow the identification of single/multiple duplication events involving stand-alone genes, cluster of genes, and/or operons or parts thereof. The identification of the function performed by the duplicated genes might reveal the existence of genes particularly prone to duplication. In addition to this, and assuming that the higher the degree of amino acid sequence identity between two proteins, the more recent the duplication event responsible for the origin of the two paralogous encoding genes, it should be possible to establish a sort of diachrony (a temporal scan) of the duplication events. Similarly to Dagan et al. [12] and, later, to Halary et al. [8] and Tamminen et al. [7], this allows interpreting the resulting networks under a molecular clock-based assumption. In the present context, proteins with 95% identity were considered more recently shared than those with 70%. Therefore, networks construction was reiterated at different sequence identity thresholds, ranging from ≥40% up to 100%.

### 3.1. Intraplasmid Network Analysis

**Figure 1:**
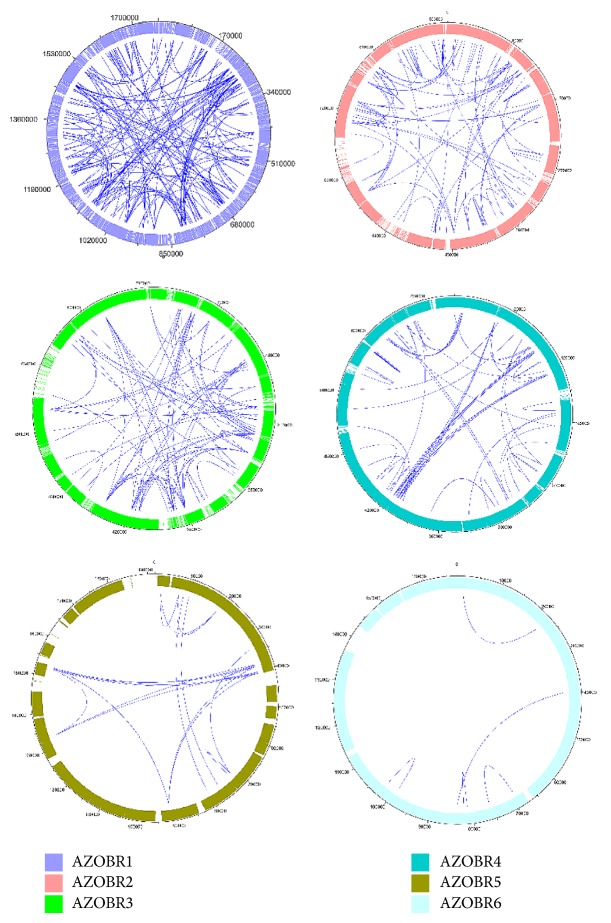
Identity based networks at intraplasmids level. All the proteins encoded by the same plasmid (nodes) are circularly arranged and are linked to the others according to their identity value. The resulting pictures for identity threshold corresponding to ≥40% are shown.

To gain a deeper understanding of the evolutionary relatedness among plasmid encoded proteins, intraplasmid networks were obtained at different thresholds (from ≥40% identity upwards 100%, always with a sequence alignment length ≥70%). The networks obtained for the six plasmids at the identity threshold of ≥40% are shown in Figure 1, whose analysis revealed the existence of paralogous genes (in-paralogs) in each of the six plasmids. Some of these genes underwent multiple duplication events. As it might be expected, the higher the plasmid size, the higher the number of nodes and links.

**Figure 2:**
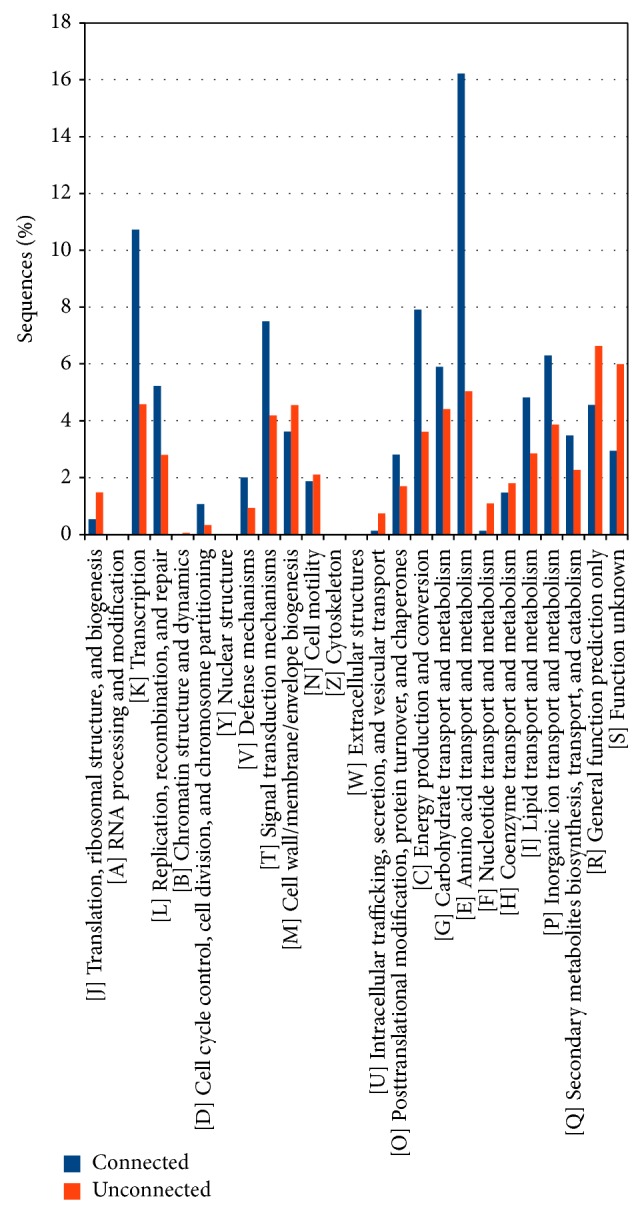
Functional characterization of the proteins coded by the plasmids of* Azospirillum brasilense* Sp245 according to the Clusters of Orthologous Groups (COG) database. The proteins are divided according to whether they have paralogous sequences within the same plasmid (connected) or not (unconnected).

Results concerning the function performed by the* A. brasilense *Sp245 plasmid encoded proteins are reported in Figure 2, showing the classification of both connected (paralogous) and unconnected proteins according to the COG database. A disproportion between the two classes was found especially for COG categories E (amino acid transport and metabolism), K (transcription), L (replication, recombination, and repair), and T (signal transduction mechanisms) suggesting a nonrandom duplication events (or duplicated gene retention) pattern for genes coding for basal biological functions and not only transposases or other mobilization related genes as previously reported [5, 9]. The analysis of the distribution of the various COG categories for each plasmid did not show any significant deviation from this common trend.

### 3.2. The Connected Component of* A. brasilense* Sp245 Plasmidome

The most important features of each* A. brasilense* Sp245 plasmid are summarized in Table 1 that include the length of each molecule, the GC mean content, and the total number of ORFs.  Table 2 shows also which proteins are connected to other proteins (in the plasmidome of* A. brasilense*) and the proportion of connected sequences on the total. In Supplementary file 2 other data are reported for a more complete picture: the predicted function of the proteins, the Preferential Organismal Sharing (POS), and the identity percent and *E*-value of the match.

**Figure 3:**
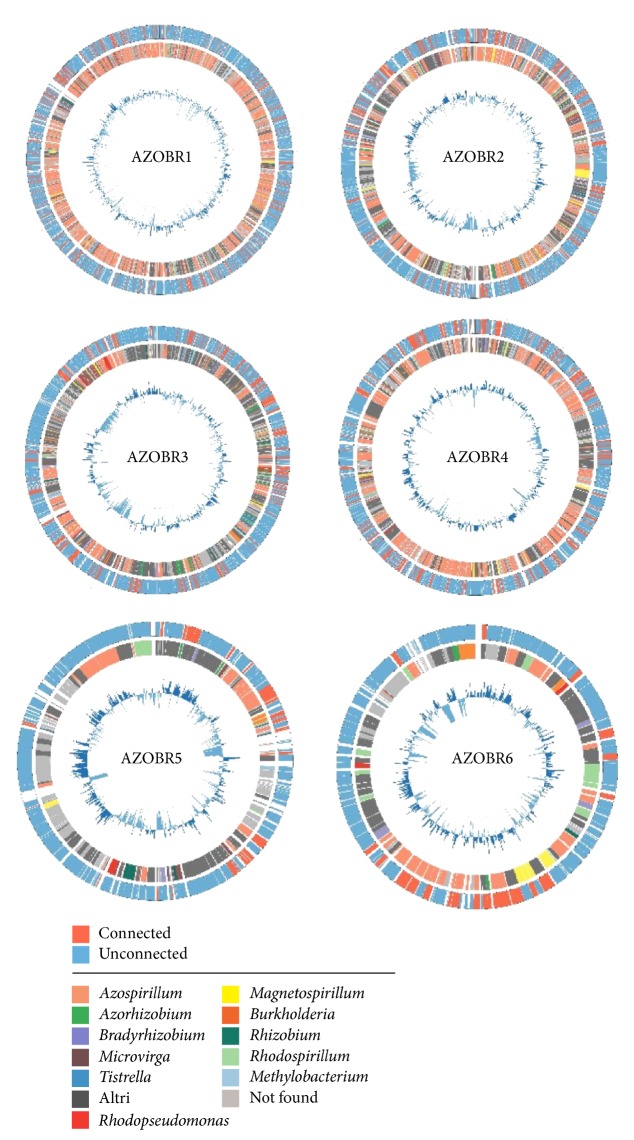
Schematic representation of the six* Azospirillum brasilense* Sp245 plasmids. The outer circle embeds connected (in- and out-paralogs) and unconnected genes, whereas the inner one corresponds to the POS. The histograms at the centre represent the GC content deviation from the mean value.

The whole body of these data is visualized in Figure 3, where the gene features are easily depicted; the outer circle discriminates between connected (red) (i.e., the genes which have been found to have paralogs on the rest of the genome) and unconnected (blue) genes. There are 244 (14.0%), 169 (19.1%), 147 (18.2%), 136 (20.3%), 29 (17.9%), and 21 (16.8%) connected proteins in AZOBR1, AZOBR2, AZOBR3, AZOBR4, AZOBR5, and AZOBR6, respectively.

From Figure 3 it is clear that no significant clusters of contiguous connected genes are present. The only exception is represented by the smallest plasmid, AZOBR6, but its tiny size makes it unsuitable for a general statistics.

The deviation of the GC content from the molecule's mean (shown by the inner histogram) is another feature that is interesting to focus on. It is expected for a plasmid [19], to be quite heterogeneous, but it changes according to the presence or absence of a coding region in the observed portion of the molecule and to its POS. In the molecule portions where no gene has been found, the GC content dramatically decreases. As for the POS, we can see, albeit with minor exceptions, that the deviation is larger when the strain with the most similar gene is not phylogenetically closely related to* A. brasilense*.

**Figure 4:**
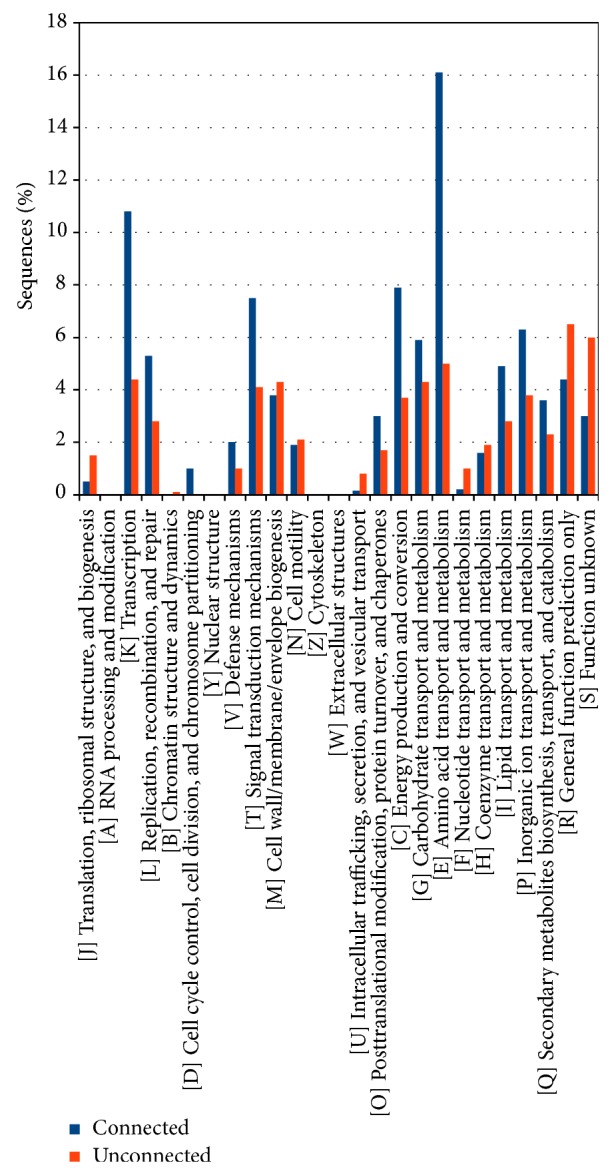
Functional characterization of the proteins coded by the plasmids of* Azospirillum brasilense* Sp245 according to the Clusters of Orthologous Groups (COG) database. The proteins are divided according to whether they have paralogous sequences within the plasmidome (connected) or not (unconnected).

An* in silico* characterization of connected and not connected genes (at intra- or interplasmid level) according to the COG database was made, and the results are reported in Figure 4. Connected and not connected proteins were annotated separately, allowing observing that some functional categories are equally represented in two classes, while in others there is a strong predominance of the connected ones, as in category K (Transcription), T (Signal transduction mechanisms), C (Energy production and conversion), and E (Amino acids transport and metabolism).

### 3.3. The Proteins Network of* A. brasilense* Sp245 Plasmidome

**Figure 5:**
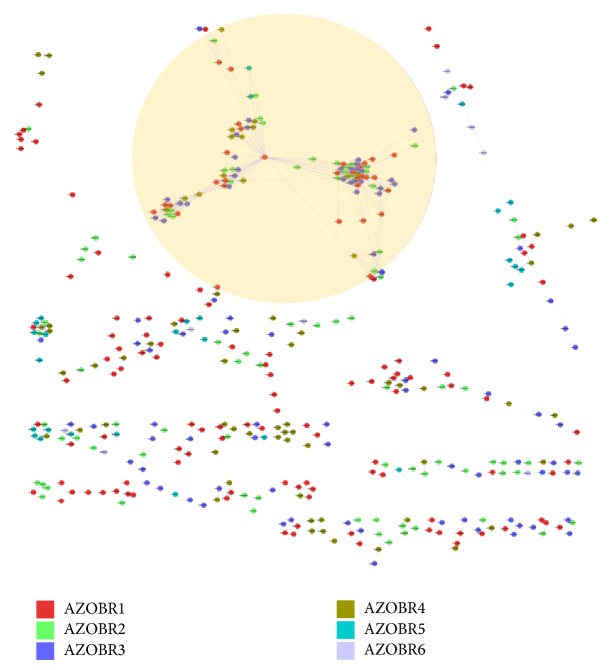
The network formed by the connected sequences (identity higher than 40% over at least the 70% of sequence) of* Azospirillum brasilense* Sp245. Each color represents a different plasmid; each node is a single protein. The biggest network component, analyzed in detail, is highlighted in yellow.

The network of connected components (i.e., in- and out-paralogs) of plasmids is represented in Figure 5. For clarity purposes, groups embedding less than 4 proteins have been omitted. The analysis of Figure 5 revealed the existence of several small networks embedding few sequences; however, some major network components including several proteins coming from the same plasmid (in-paralogs) and/or different ones (out-paralogs) were also detected. Data obtained revealed that most components, especially the larger ones, were constituted by in- and/or out-paralogs, even though few components were constituted only by in-paralogs.

**Figure 6:**
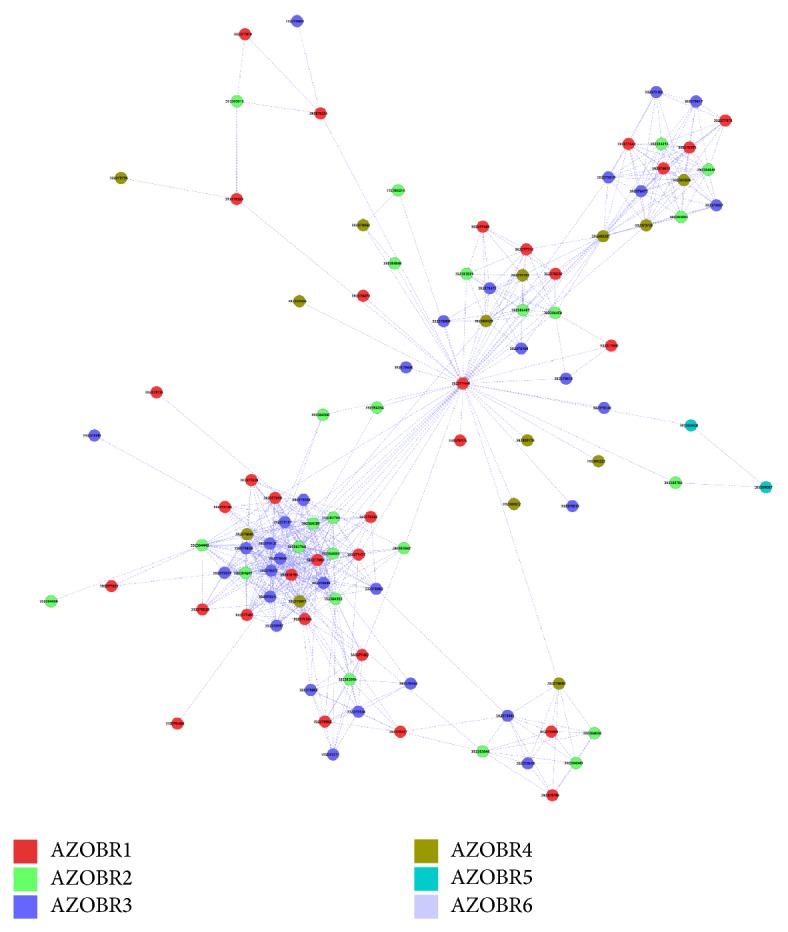
Details of the largest network component identified. All the genes code for proteins that belong to the ABC transporters family. The shape of the network is slightly different from the one shown in Figure 5 due to the algorithm used by the software, which automatically arranges the nodes to fit the available space.

**Figure 7:**
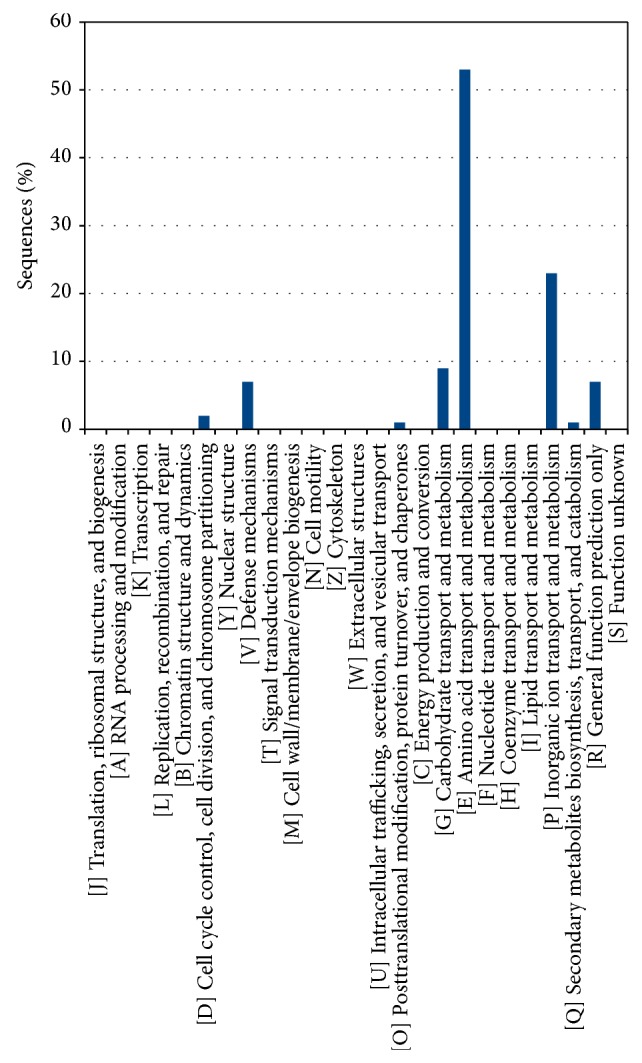
Functional characterization of the proteins coded by the genes of the largest network component found amongst the plasmidome connected proteins.

A magnification of the largest network component, including 104 proteins encoded from five* A. brasilense* Sp245 plasmids (32, 25, 31, 14, and 2 from AZOBR1, AZOBR2, AZOBR3, AZOBR4, and AZOBR5, resp.), is reported in Figure 6. This cluster embeds proteins encoded by both in- and out-paralogs and it revolves around a small protein (44 amino acids) with an unknown function. Proteins belonging to this cluster are listed in Table 2 along with their putative function. These proteins were separately characterized according to the COG database, with the results shown in Figure 7. Data obtained revealed that the categories containing almost totality of proteins are those related to the ABC superfamily involved in the transport of amino acids, inorganic ions, and carbohydrates.

For the proteins belonging to the plasmids, the identification of the POS was carried out. Data obtained are reported in Figure 3. Another feature that, less predictably, changes with the size, is the POS of the genes (inner circle). Most of the genes of the first—and largest—plasmid AZOBR1 have as POS* A. lipoferum*, which has been shown [4] being the phylogenetically nearest organism to* A. brasilense*, while, with the decrease of plasmids size, more and more strains are featured, as can be seen visually by the mosaic-like structure of the inner circle.

### 3.4. Plasmids versus Chromosome Network Analysis

**Figure 8:**
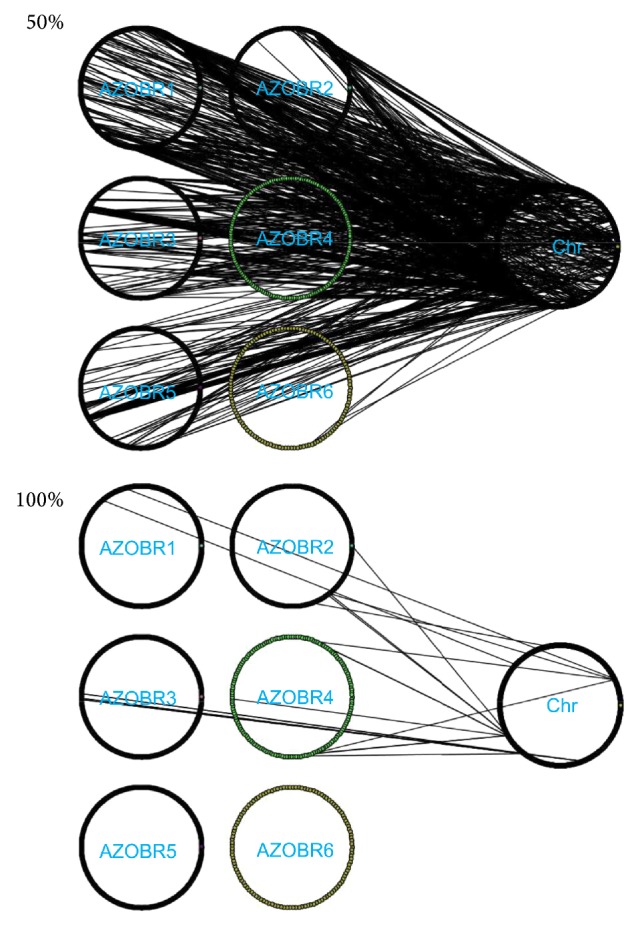
Evolutionary relationships among the* A. brasilense* Sp245 plasmids and chromosome at a similarity threshold of 50% and 100%. Each link connects a protein of the chromosome with a homologous one on a plasmid.

**Figure 9:**
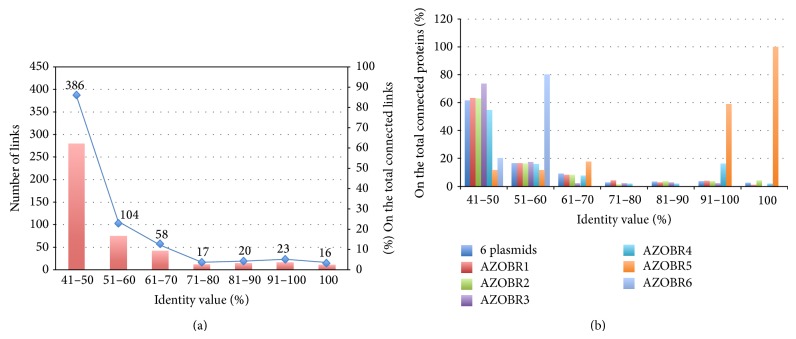
Histograms classifying the connected sequences of the plasmids according to the identity percent with their most similar sequence found within the genome. In the upper part (a) a line shows the total number of sequences with an identity equal to or greater than the one of each category. The graph (b) discriminates among different plasmids.

The networks constructed to analyze the evolutionary relationships among the* A. brasilense *Sp245 plasmids and chromosome were constructed; two of them (at an identity threshold of ≥50% and 100%) are shown in Figure 8 whereas the entire set of networks constructed at ≥40%, ≥50%, ≥60%, ≥70%, ≥80%, ≥90%, and =100% is reported in Supplementary file 3. The analysis of the networks showed that each plasmid shared some genes with the* A. brasilense* Sp245 chromosome, even though their number is variable. Overall, as expected, the higher the plasmid size is, the higher the number of links and genes shared with the chromosomes is. A further analysis (Figure 9) revealed a progressive decrease of links number (bars representing their absolute number and the line the % of links with an identity equal or grater of the considered range) with the increase of identity thresholds, with an exception represented by the AZOBR5 plasmid, in which we observed an increase of the connected component at 91–99% identity threshold; this result can be interpreted as a peak of recent duplication events inside this plasmid. Nevertheless, the proportion of high identity connections (≥71%) among plasmids and chromosomes remains fairly constant and does not reach zero, suggesting that recent molecular evolutionary events (plasmid-chromosome cross talk) took place. The same plot (Figure 9(b)) highlights that a high relative abundance of proteins sharing a high degree of sequence similarity is not a general trend for all plasmids but is related, in this case, to the molecular evolutionary event occurring in plasmids 4 and 5; this finding strongly suggests that plasmids inside the same genome can follow different evolutionary trajectories.

**Figure 10:**
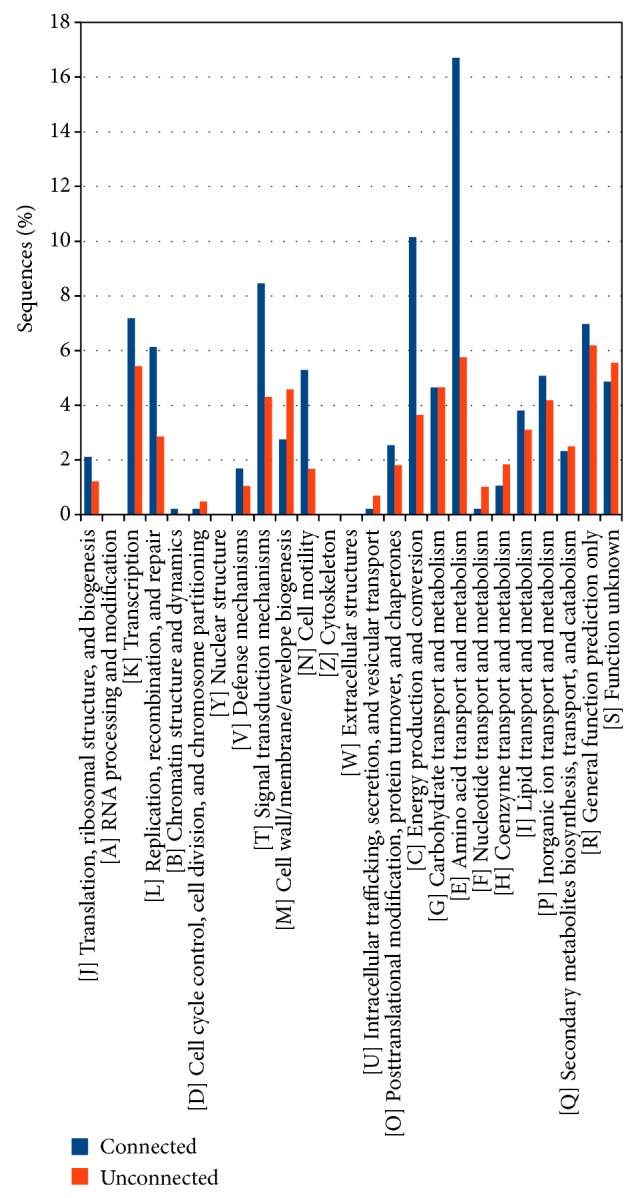
Functional characterization of the proteins coded by the plasmids of* Azospirillum brasilense* Sp245 according to the Clusters of Orthologous Groups (COG) database. The proteins are divided according to whether they have paralogous sequences on the chromosome (connected) or not (unconnected).

COG annotation of connected and unconnected proteins (Figure 10) shows again a disproportion not only for categories E, K, T, and L (as for intraplasmids proteins) but also for categories C (Energy production and conversion) and N (Cell motility). Supplementary file 4 reports in detail the proteins of the two groups (i.e., connected and unconnected) according to the COG categories they have been assigned.

## 4. Discussion

The main results of the* Azospirillum *plasmidome analysis are shown in Figure 3: the absence of a cluster-like organization of the connected genes is immediately clear. On the contrary, they are scattered throughout the molecule. The inner circle, representing the POS of the proteins, allows some interesting observations. In the biggest plasmid, AZOBR1, genes show strong homology almost exclusively from other strains of the genus* Azospirillum* (orange), especially* A. lipoferum *4B (see also Supplementary file 2). With the decrease in size of the molecule, the POS becomes more and more varied. Observing the organisms whose proteins are homologous to those of* A. brasilense *(Supplementary file 2), it can be noted that there are no clusters of genes related to the same strain, exception made for* A. lipoferum*. This could lead to the supposition that no particular clusters of genes are involved in horizontal transfer with distantly related strain. To support this hypothesis, it can be useful to look at the GC content since compositional features can be used to trace back possible HGT events [19]: indeed, some blocks in the inner circle correspond to a noticeable deviation from the mean value in GC content (Figure 3). This is a strong hint that suggests the actual—and relatively recent—origin of the cluster from another organism and not just a common ancestral origin of the sequences. Even if, only with these data, it is not possible to unambiguously determine whether these cluster are effectively inherited from a horizontal transfer or not, the abrupt variations in GC content witness for this hypothesis.

Concerning the intraplasmid network presented in Figure 1, it can be readily observed that with the decrease in size of the molecule, the connections decrease, and not only their absolute number but also their density decreases. Concerning the physical organization of the duplicate genes, no particular clusters of connected genes can be seen at intramolecule level.

The graphs classifying the connected plasmid sequences with the chromosome according to their identity percentage (Figure 9) show that the majority of proteins have a low identity (40–50%) with their connected sequences. This could mean that the event that led to the replication (e.g., an HGT or the effect of a transposase) occurred not recently, and the sequences had the time to undertake sequence divergence, eventually coding for related but not identical proteins. Nevertheless there are some interesting differences among the plasmids: the diachrony of gene duplications event, assuming that proteins with a strong sequence similarity are the outcome of (relatively) recent duplication, is not identical for all the plasmids, suggesting that replicons inside the same cytoplasm might have different evolutionary models.

As for the COG characterization of the proteins (Figure 10), divided into connected and not connected, the results show a strong predominance of connected proteins in the categories regarding the transporters (category E), followed by the proteins involved in the energy production and conversion (category C), and to a lesser extent by those involved in the signal transduction mechanisms (category T). These findings are in agreement with those of previous studies investigating the HGT in* Azospirillum* genus [4]. In order to investigate more thoroughly the evolutionary relationships among the* A. brasilense* plasmids encoded proteins we focused our attention on the connected components of the overall network built in the first stages of this analysis.

In Figure 5, which shows all the connected components with at least 4 sequences, we can immediately see that the connected nodes, especially in the bigger networks, belong to different plasmids. Clusters of paralog proteins are therefore rare, if not absent, inside the same molecule. This suggests an intense activity of rearrangement and horizontal transfers among plasmids, rather than a preferred cross talk of each plasmid inside itself.

From Figure 5 it is possible to see that there is a big network component with nodes that are heavily connected to each other. Components belonging to different plasmids are coloured differently. This network is shown in detail in Figure 6, and the functional annotations of the 104 proteins that belong to it are shown in Table 2. Their COG classification is shown in Figure 7. As we can see from these data, this network component is made of proteins mainly related to the superfamily of ABC transporters. The COG characterization is particularly clear, for the three functional groups almost all proteins belong to are closely related to the ABC superfamily. The amino acid transport function is predominant (50% of all the sequences), followed by the inorganic ion transport. These proteins can be related to some kind of antidrug resistance or to the exchange of molecules with the plants* Azospirillum* associates to. As with the other networks components, also here we can see the tendency to have nodes scattered through all the plasmids (exception made for AZOBR6, the smallest one), without particular differences among them, thus confirming the general observation of an intense exchange of genes among plasmids.

Finally, analyzing the connections of the plasmids versus the chromosome (Supplementary file 3) the idea that different replicon types (i.e., plasmids and chromosomes) inside the same cytoplasm may follow different evolutionary trajectories showing a different level of trade-off and reshuffling with the chromosome genome is reinforced.

## 5. Conclusions

The analyses performed in this work shed more light on the plasmidome of* Azospirillum* Sp245, which plays an important role in many ecosystems. Duplication events shaped the architecture of* A. brasilense* replicons as highlighted by the presence of paralogous genes in other parts of the plasmidome, usually on a different plasmid. From this we supposed an intense activity of gene exchange among plasmids residing in the same cell. We looked for the POS (Preferential Organismal Sharing—that is, the strongest evolutionary relatedness of each gene in the replicons with other microorganisms) and found out that the taxonomical proximity is more important than the sharing of the same ecological niche, for most of the POS were related to* A. brasilense* close species* Azospirillum lipoferum*.

Interestingly, both the intraplasmid and plasmid versus chromosome networks highlight that paralogous gene families may show a different distribution in identity thresholds categories in the different plasmids. This finding strongly suggests that different replicons inside the same cytoplasm may follow unequal molecular evolution processes: in this specific study case, plasmids AZOBR4 and AZOBR5 (the smallest, with the exception of AZOBR6, a very small plasmid for which inferences are hard to draw) are enriched in high identity (recently formed) paralogous gene families.

A functional annotation of the connected proteins revealed that a large set of them belongs to the ABC superfamily, primarily involved in the transport of amino acids. This may suggest the presence of an evolutionary trend in the (horizontal) sharing of such genes in this particular organism.

Finally, in this work a simple yet effective bioinformatics pipeline for the network-oriented investigation of informative molecule was presented, allowing also scientists lacking a strong bioinformatic background to perform the analyses presented herein.

## Supplementary Material

Supplementary File 1: Pipeline followed to find the connected proteins coded by the Azospirillum brasilense plasmidome and to characterize their function.
Supplementary File 2: Predicted function, Preferential Organismal Sharing (POS), and identity percent of the first BLAST match for each connected protein of the Azospirillum brasilense plasmidome.
Supplementary File 3: Plasmids – Chromosome connections at different identity thresholds, from 40% to 100%.
Supplementary File 4: List of all proteins coded by the Azospirillum brasilense plasmidome, with their functional characterization according to the COG (Cluster of Orthologous Groups) database.

## Figures and Tables

**Table 1:** List of *A. brasilense* plasmid and encoded proteins. Threshold for connected sequences: >40% identity over at least the 70% of the sequence length. Connections considered are both intra- and interplasmids and those with the chromosome. Connected percent is on the total proteins of each plasmid.

Plasmid	Length (bp)	GC (%)	Proteins encoded			
			Total number	Unconnected	Connected	% Connected
AZOBR1	1766028	68.6	1748	1504	244	13.96
AZOBR2	912449	68.3	884	715	169	19.12
AZOBR3	778798	68.2	808	661	147	18.19
AZOBR4	690334	68.9	671	535	136	20.27
AZOBR5	191828	66.7	162	133	29	17.90
AZOBR6	167364	66.8	125	104	21	16.80

**Table 2:** List of *A. brasilense* Sp245 plasmid-borne paralogous genes and putative encoded function.

Plasmid	GI	Function encoded
AZOBR1	392377419	Putative spermidine/putrescine ABC transporter (ATP-binding protein)
AZOBR1	392377442	Branched-chain amino acid ABC transporter, ATP-binding component (LivF-like) (fragment)
AZOBR1	392377443	Branched-chain amino acid ABC transporter, ATP-binding component (LivG-like) (modular protein)
AZOBR1	392377444	Protein of unknown function
AZOBR1	392377462	Amino acid ABC transporter, ATP-binding component
AZOBR1	392377476	Glycine betaine/choline ABC transporter, ATP-binding component
AZOBR1	392377481	Putative spermidine/putrescine ABC transporter subunit, ATP-binding component
AZOBR1	392377539	Putative ABC transporter (ATP-binding subunit)
AZOBR1	392377651	ATP-binding protein, ABC-type iron transporter (fragment)
AZOBR1	392377679	Putative branched-chain amino acid transport system, ATP-binding protein
AZOBR1	392377680	Putative branched-chain amino acid transport system, ATP-binding protein
AZOBR1	392377710	Putative branched-chain amino acid ABC transporter, ATP-binding component (livG-like)
AZOBR1	392377978	Iron-hydroxamate ABC transporter, ATP-binding component
AZOBR1	392377992	Putative ABC transporter (ATP-binding protein)
AZOBR1	392378002	Glutamine ABC transporter, ATP-binding component
AZOBR1	392378029	Putative sugar ABC transporter (ATP-binding protein), with TOBE domain (C-terminal)
AZOBR1	392378103	Nitrate transporter ATPase
AZOBR1	392378153	Putative ABC transporter, ATP-binding protein
AZOBR1	392378175	Putative ABC transporter, ATP-binding component
AZOBR1	392378223	Iron-dicitrate ABC transporter, ATP-binding component
AZOBR1	392378234	Iron-dicitrate transporter subunit, ATP-binding component of ABC superfamily, KpLE2 phage-like element
AZOBR1	392378265	Sulfate/thiosulfate ABC transporter, ATP-binding component
AZOBR1	392378307	Methionine ABC transporter, ATP-binding component
AZOBR1	392378373	Putative branched-chain amino acid ABC transporter, ATP-binding component (livF-like)
AZOBR1	392378406	Cell division ATP-binding protein
AZOBR1	392378473	Chromosome segregation protein SMC
AZOBR1	392378633	High-affinity branched-chain amino acid ABC transporter (ATP-binding protein)
AZOBR1	392378634	ABC transporter ATP-binding protein
AZOBR1	392378646	Putative ABC transporter (ATP-binding protein)
AZOBR1	392378736	Putative ABC transporter, ATP-binding protein
AZOBR1	392378799	Dipeptide ABC transporter, ATP-binding component
AZOBR1	392378800	Dipeptide ABC transporter, ATP-binding component
AZOBR2	392383686	Branched-chain amino acid ABC transporter, ATP-binding component
AZOBR2	392383691	Branched-chain amino acid ABC transporter, ATP-binding component
AZOBR2	392383703	Type I secretion, ATP-binding protein
AZOBR2	392383786	ABC transporter, ATP-binding component (tauB-like)
AZOBR2	392383794	ABC transporter, ATP-binding component
AZOBR2	392383844	Dipeptide ABC transporter, ATP-binding component
AZOBR2	392383919	Iron-dicitrate transporter subunit, ATP-binding component of ABC superfamily, KpLE2 phage-like element
AZOBR2	392383942	Glycine betaine/L-proline transport, ATP-binding protein proV
AZOBR2	392383954	Putative amino acid transport protein, ATP-binding protein
AZOBR2	392384040	ABC transporter (ATP-binding protein)
AZOBR2	392384049	Oligopeptide transport protein (ABC superfamily, ATP-binding protein)
AZOBR2	392384050	Oligopeptide transport protein (ABC superfamily, ATP-binding protein)
AZOBR2	392384088	Putative spermidine/putrescine ABC transporter (ATP-binding protein)
AZOBR2	392384214	Putative ABC transporter (ATP-binding protein)
AZOBR2	392384247	Putative ABC transporter (ATP-binding protein)
AZOBR2	392384353	Putative sulfonate ABC transporter, ATP-binding protein (ssuB/tauB/ycbE-like) (fragment)
AZOBR2	392384354	Aliphatic sulfonates import, ATP-binding protein ssuB (fragment)
AZOBR2	392384385	Putative ABC transporter (ATP-binding protein)
AZOBR2	392384417	Putative lipopolysaccharide transport protein B, ATP-binding component of ABC superfamily
AZOBR2	392384418	Putative Branched-chain amino acid ABC transporter (ATP-binding protein), livF-like protein
AZOBR2	392384442	Putative sugar ABC transporter (ATP-binding protein), with TOBE domain (C-terminal)
AZOBR2	392384449	Branched-chain amino acids ABC transporter, ATP-binding component
AZOBR2	392384450	Branched chain amino acids ABC transporter, ATP-binding component
AZOBR2	392384505	Putative ATP-binding protein of sugar ABC transporter
AZOBR2	392384506	Putative ATP-binding protein of sugar ABC transporter
AZOBR3	392378942	Oligopeptide transport protein (ABC superfamily, ATP-binding protein)
AZOBR3	392378943	Dipeptide ABC transporter, ATP-binding component
AZOBR3	392378957	Putative Spermidine/putrescine ABC transporter (ATP-binding protein)
AZOBR3	392378962	ABC transporter related protein
AZOBR3	392378982	Glutamine ABC transporter, ATP-binding component
AZOBR3	392379006	ABC transporter, permease protein (N-ter), ATP-binding protein (C-ter) putative branched-chain amino acid transport protein
AZOBR3	392379007	High-affinity branched-chain amino acid ABC transporter (ATP-binding protein)
AZOBR3	392379018	Putative ABC tranpsorter, permease protein (N-ter) and ATP-binding protein (C-ter), branched-chain amino acid transport protein
AZOBR3	392379019	High-affinity branched-chain amino acid transport protein (ABC superfamily, ATP-binding)
AZOBR3	392379046	Spermidine/putrescine import ATP-binding protein potA
AZOBR3	392379104	Putative branched-chain amino acid ABC transporter, ATP-binding protein
AZOBR3	392379105	Putative branched-chain amino acid ABC transporter, ATP-binding protein
AZOBR3	392379134	Putative amino acid transport protein, ATP-binding protein
AZOBR3	392379137	ABC transporter, ATP-binding component
AZOBR3	392379167	Alkanesulfonate ABC transporter, ATP-binding protein
AZOBR3	392379193	Macrolide ABC transporter, ATP-binding and permease domains
AZOBR3	392379260	Putative ABC transporter (ATP-binding protein) (tauB-like)
AZOBR3	392379271	Putative ABC transporter (ATP-binding protein)
AZOBR3	392379302	Putative ABC transporter (ATP-binding protein)
AZOBR3	392379311	Putrescine transport protein (ABC superfamily, atp_bind)
AZOBR3	392379334	Putative ABC transporter (protein fusion consisting of two ATP-binding domains and permease)
AZOBR3	392379338	ABC transporter ATP-binding protein
AZOBR3	392379404	Copper ABC transporter, ATP-binding component
AZOBR3	392379472	ABC tranpsorter, permease protein (N-ter), ATP-binding protein (C-ter), putative branched-chain amino acid transport protein
AZOBR3	392379473	ABC transporter, ATP-binding protein, putative branched-chain amino acid transport protein
AZOBR3	392379616	Putative branched-chain amino acid transport system, ATP-binding protein
AZOBR3	392379617	Putative branched-chain amino acid transport system, ATP-binding protein
AZOBR3	392379643	Phosphonates import ATP-binding protein phnC (ABC superfamily, atp_bind)
AZOBR3	392379659	Putative ABC transporter (ATP-binding protein)
AZOBR3	392379666	Hemin ABC transporter, ATP-binding component
AZOBR3	392379711	Putative amino acid transport protein, ATP-binding protein
AZOBR4	392379725	High-affinity branched-chain amino acid transport protein (ABC superfamily, ATP-binding)
AZOBR4	392379726	High-affinity branched-chain amino acid transport protein (ABC superfamily, ATP-binding)
AZOBR4	392379756	Protein of unknown function
AZOBR4	392379885	Putative spermidine/putrescine transport protein (ABC superfamily, atp_bind)
AZOBR4	392379891	Putative dipeptide/oligopeptide/nickel ABC transporter, ATP-binding protein
AZOBR4	392379956	Putative phosphonate ABC transporter, ATPase component
AZOBR4	392379971	Putative spermidine/putrescine transport protein (ABC superfamily, atp_bind)
AZOBR4	392380065	Sugar ABC transporter, ATP-binding component (RbsA-like)
AZOBR4	392380072	Sugar ABC transporter, ATP-binding component
AZOBR4	392380170	Putative ABC transporter, fused ATP-binding/permease protein
AZOBR4	392380222	Putative ABC transporter, ATP-binding component
AZOBR4	392380307	Branched-chain amino acid ABC transporter, ATP-binding protein
AZOBR4	392380328	Branched-chain amino acids ABC transporter, ATP-binding component
AZOBR4	392380329	Branched chain amino acids ABC transporter, ATP-binding component
AZOBR5	392384567	Putative secretion ATP-binding protein (ABC-type transporter family), putative toxin/protease secretion system
AZOBR5	392384634	Putative ABC transporter family, HlyB subfamily, putative protease secretion (ATP-binding protein)
