# Supplementary material for: Network Analysis of Plasmidomes: The Azospirillum brasilense Sp245 Case
Source: Int J Evol Biol. 2014 Dec 29;2014:951035. doi: 10.1155/2014/951035 (PMC4295147; doi:10.1155/2014/951035)
Supplement: Supplementary file 1 — Pipeline followed to find the connected proteins coded by the Azospirillum brasilense plasmidome and to characterize their function. Supplementary File 2: Predicted function, Preferential Organismal Sharing (POS), and identity percent of the first BLAST match for each connected protein of the Azospirillum brasilense plasmidome. Supplementary File 3: Plasmids – Chromosome connections at different identity thresholds, from 40% to 100%. Supplementary File 4: List of all proteins coded by the Azospirillum brasilense plasmidome, with their functional characterization according to the COG (Cluster of Orthologous Groups) database. [file 951035.f1.ppt]

## Slide 1
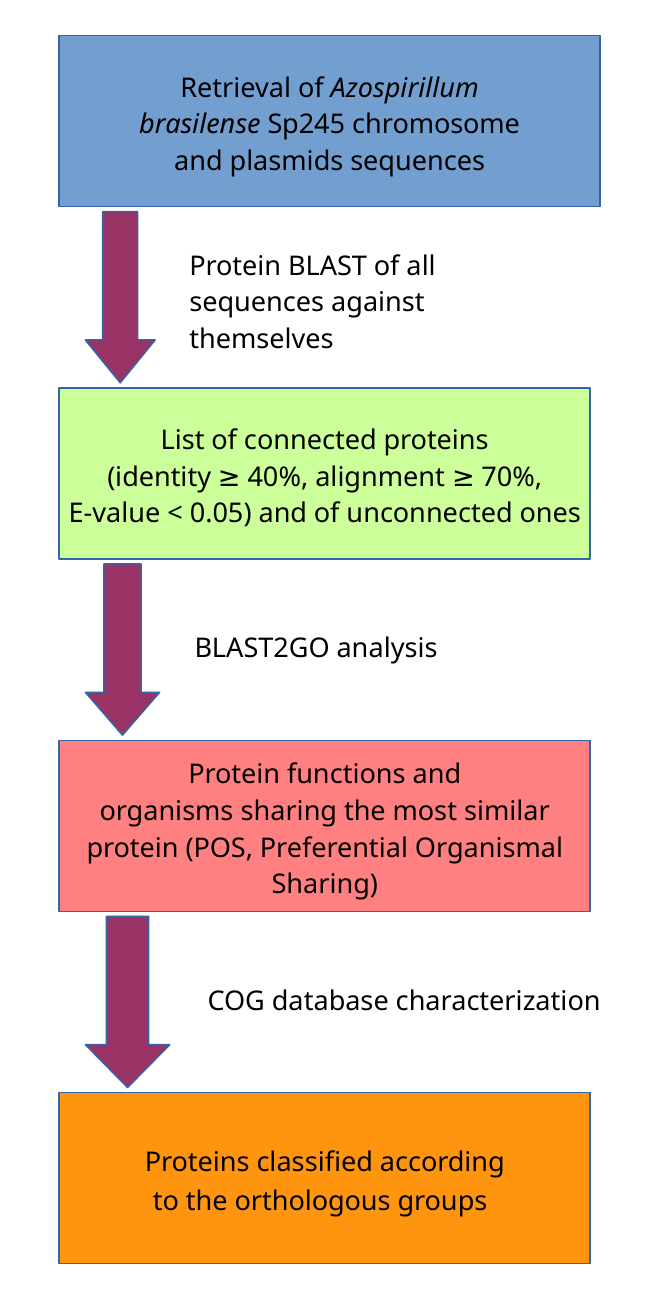

Retrieval of Azospirillum
brasilense Sp245 chromosome
and plasmids sequences
Protein BLAST of all sequences against themselves
List of connected proteins
(identity ≥ 40%, alignment ≥ 70%,
E-value < 0.05) and of unconnected ones
BLAST2GO analysis
Protein functions and
organisms sharing the most similar
protein (POS, Preferential Organismal
Sharing)
COG database characterization
Proteins classified according
to the orthologous groups
