# Supplementary material for: Network Analysis of Plasmidomes: The Azospirillum brasilense Sp245 Case
Source: Int J Evol Biol. 2014 Dec 29;2014:951035. doi: 10.1155/2014/951035 (PMC4295147; doi:10.1155/2014/951035)
Supplement: Supplementary file 3 [file 951035.f3.pptx]

## Slide 1
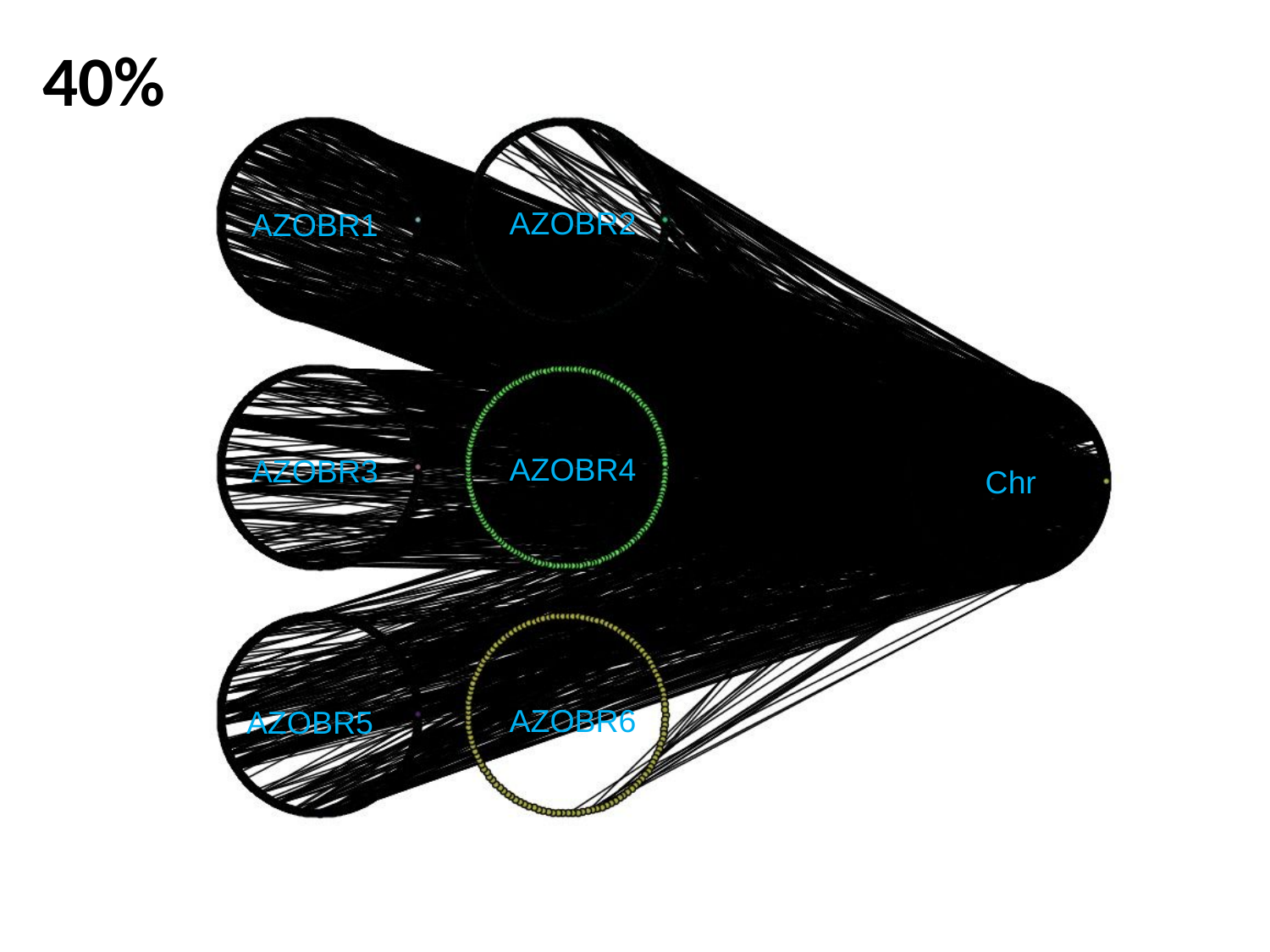

40%
AZOBR2
AZOBR1
AZOBR4
AZOBR3
Chr
AZOBR6
AZOBR5

## Slide 2
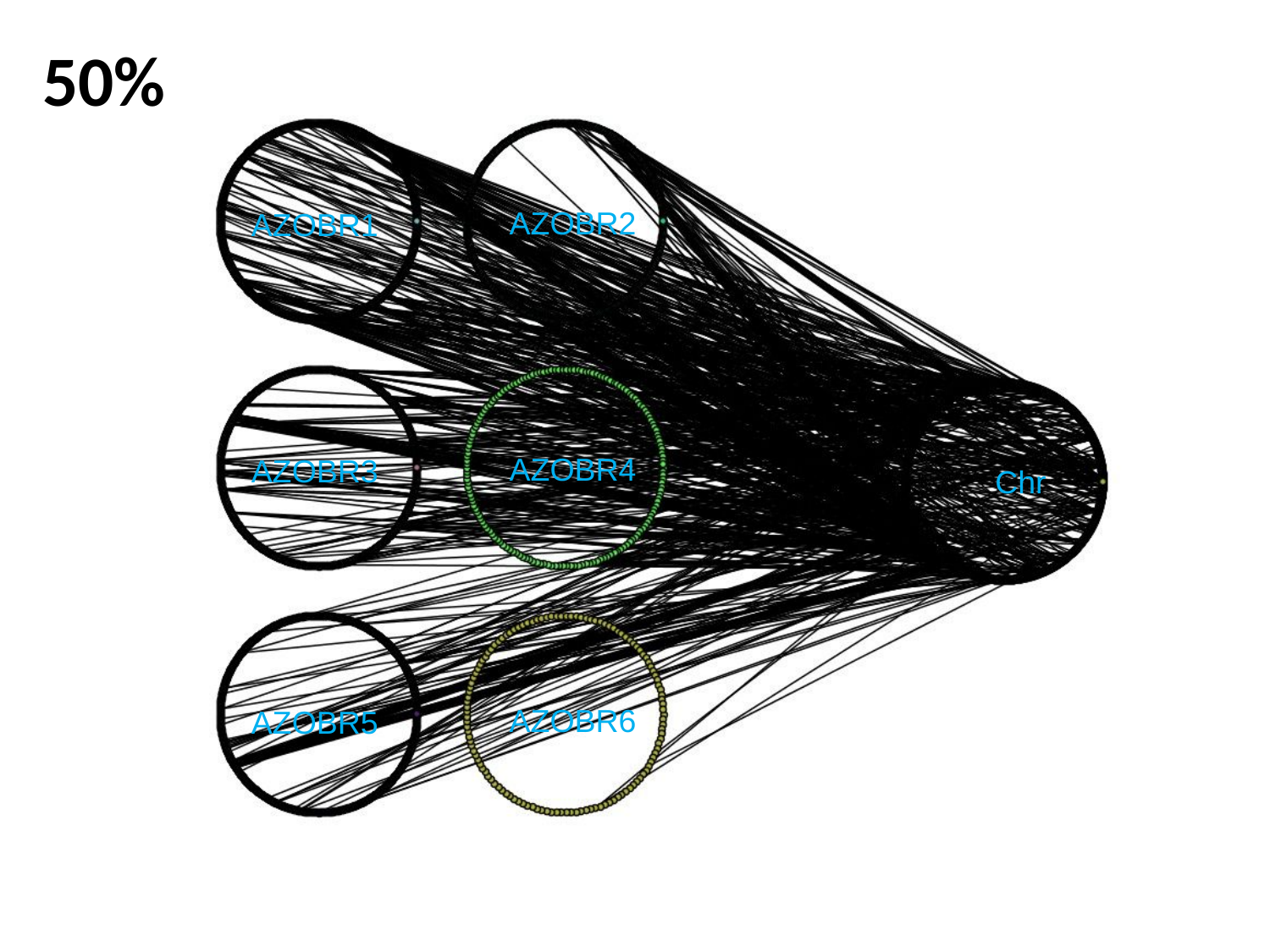

50%
AZOBR2
AZOBR1
AZOBR4
AZOBR3
Chr
AZOBR6
AZOBR5

## Slide 3
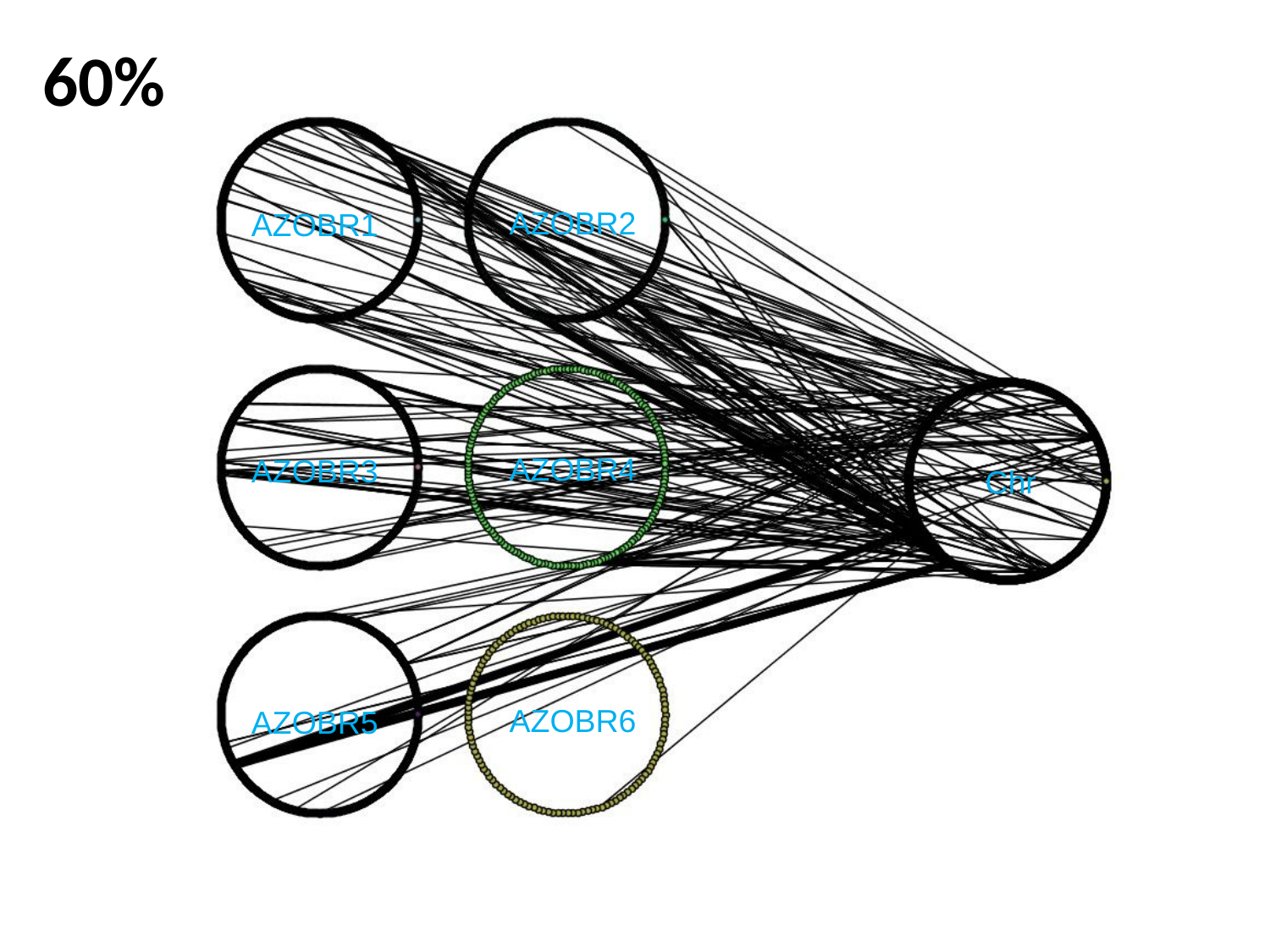

60%
AZOBR2
AZOBR1
AZOBR4
AZOBR3
Chr
AZOBR6
AZOBR5

## Slide 4
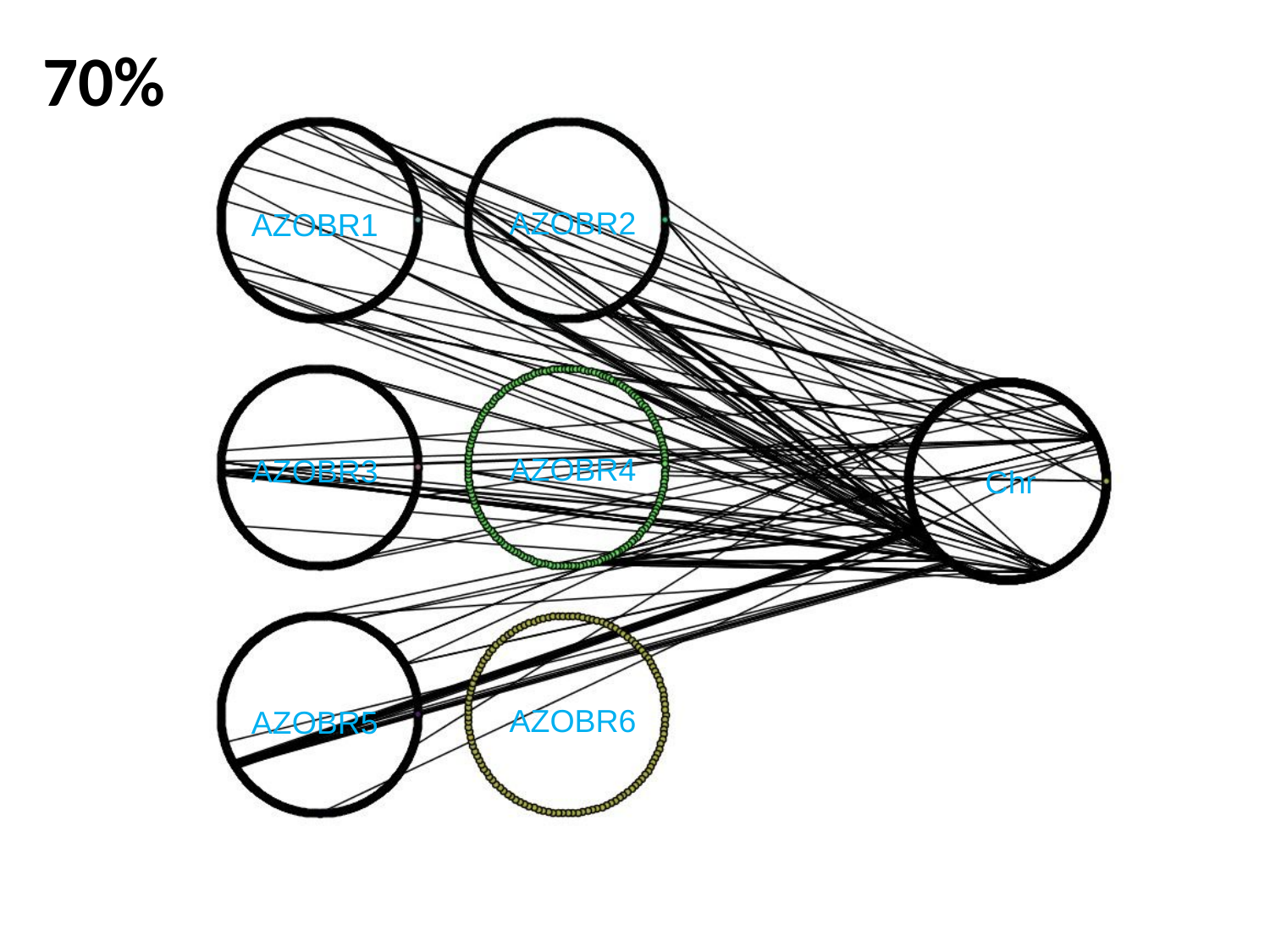

70%
AZOBR2
AZOBR1
AZOBR4
AZOBR3
Chr
AZOBR6
AZOBR5

## Slide 5
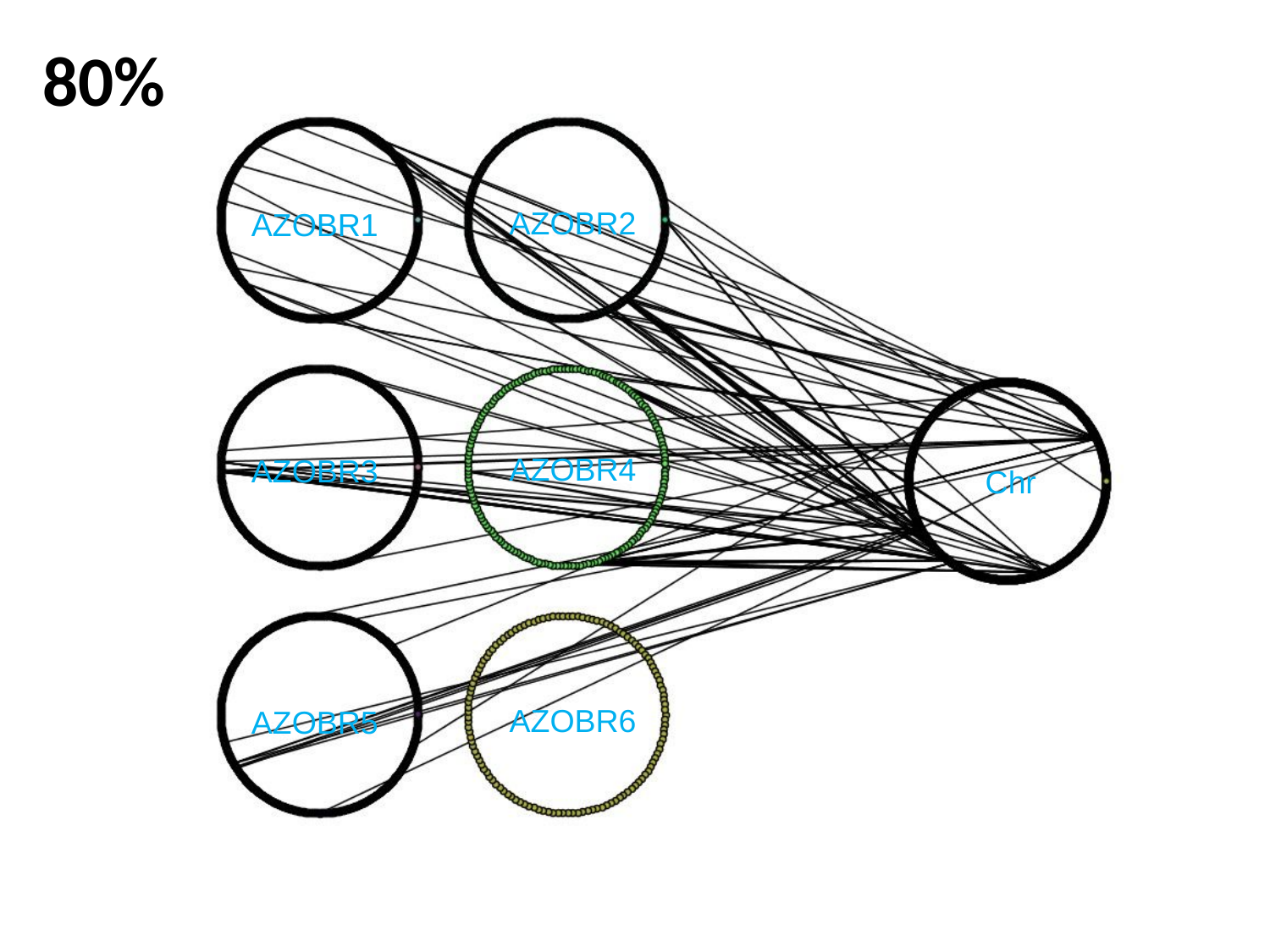

80%
AZOBR2
AZOBR1
AZOBR4
AZOBR3
Chr
AZOBR6
AZOBR5

## Slide 6
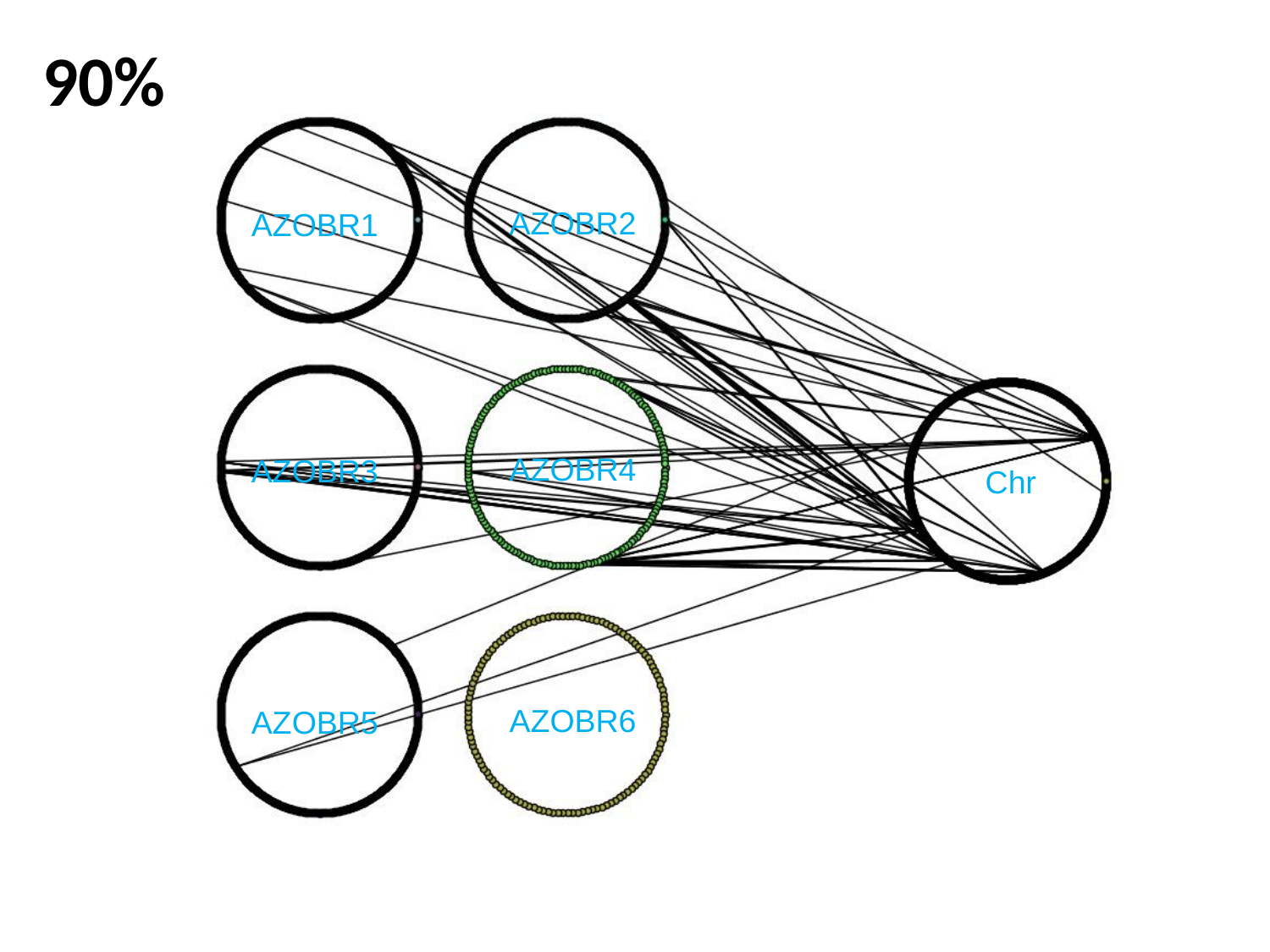

90%
AZOBR2
AZOBR1
AZOBR4
AZOBR3
Chr
AZOBR6
AZOBR5

## Slide 7
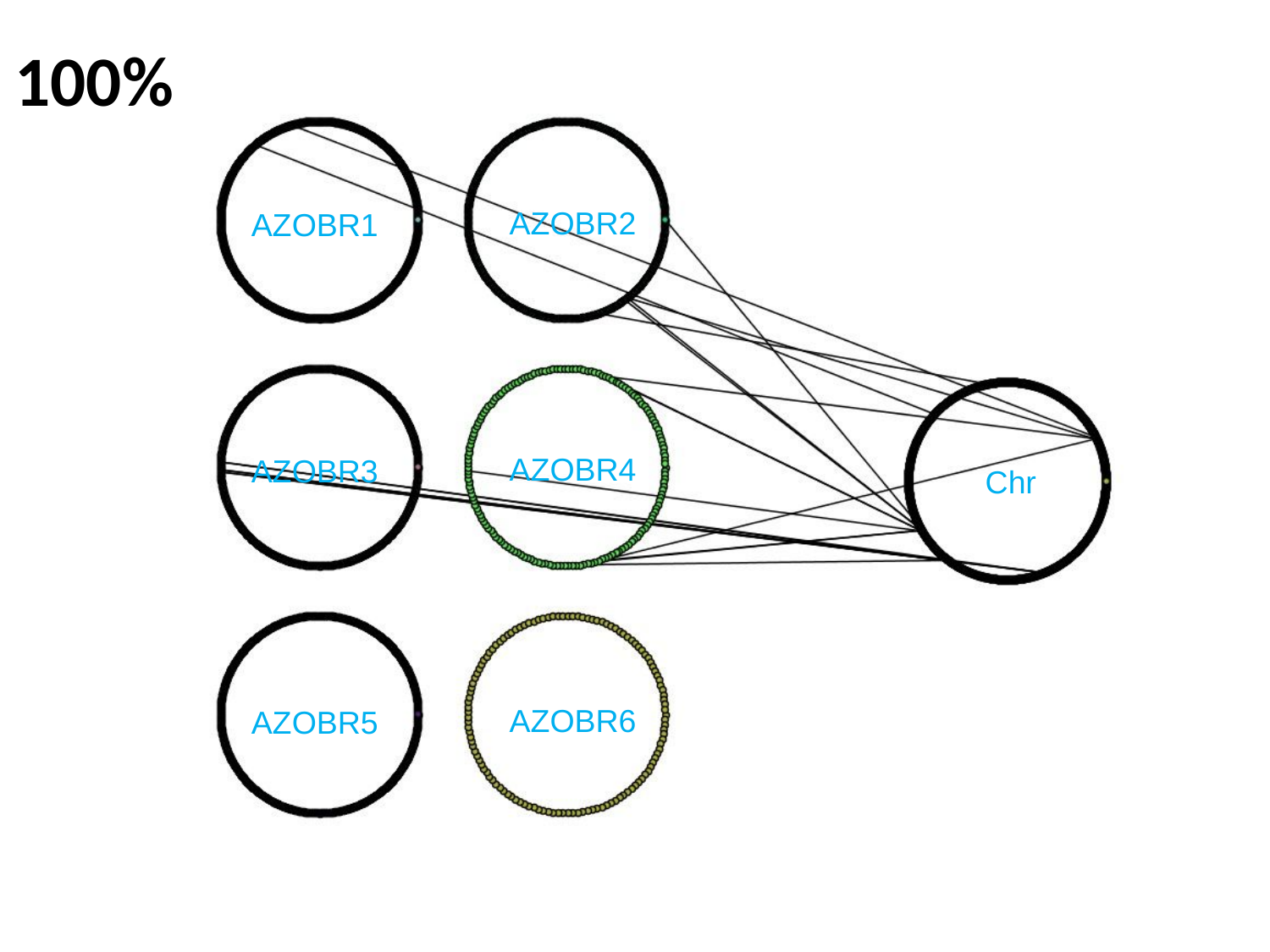

100%
AZOBR2
AZOBR1
AZOBR4
AZOBR3
Chr
AZOBR6
AZOBR5
